# EHMTI-0127. Three faces of pain-herpes zooster ophthalmicus

**DOI:** 10.1186/1129-2377-15-S1-C25

**Published:** 2014-09-18

**Authors:** A Jori Birkas

**Affiliations:** 1 Clinical Neurology, National Institute of Neurosciences, Budapest, Hungary

## Objectives

64 years old female patient’s medical history from beginning of the pain will be presented following the change and pain relief to recovery.

## Background

Herpes zoster ophthalmicus (HZO) occurs only the people who have been previously infected with varicella zooster virus (VZV). Primary infection is a chickenpox (95% VZV seropositivive in US adults). Reactivated forms its dormant status is the dorsal root, cranial nerve and other sensory ganglia may lay for years to decades. Reactivation of latens VZV localizated cutaneous rash erupting a single dermatome. It may travel along a sensory axon of the skin from vesicular lesions. Perineuritis causes intensive pain in the nerve distributionLifetime reactivation of VZV as shingles 50% incidence, HZO is rare (10-20%) of all VZV causes. HZO is a trigeminal manifestation of VZV. Reactivated VZV travels down V/1 and V/2 centripetal distribution. Contagious spread of the VZV may lead of the involvement of the other cranial nerves resulting optic neuropathy (II) isolated cranial nerve palsies (III. IV. especially IV.) neurogene motility disorders. Predisposing factors: immunecompromissed condition (decreased T-cell activity), acute herpes simplex virus infection and other reacting factors. Complications 50% of HZO patient is a postherpetic neuralgia (20% of a cases) involves the orbit (10-25% of a cases).

Acute management: antiviral agent immediately-best prognosis started early-within 72 hours and general measures, ophthalmology consultation.

Pain management: acute and postherpetic neuralgia.

Our patient evaluated for left sided facial pain, unilateral "burning eye" and malaise that began two days ago. The neurological examination has been proven partially n. oculomotorius palsy of the left side and hyperaesthesia in dermatome V/1 unilateral side.

Neuroimaging studies (cerebral and orbital CT and MRI) have shown normal status.

Two days later on the face in the V/1 and V/2 dermatome we detected special characteristic skin symptom.

## Conclusion

The diagnosis was clear: herpes zooster ophthalmicus (HZO).

We present evolution change in the nature of pain, following and demonstrating the eye movement disorders and skin signs from termination to recorvery process.

No conflict of interest.

